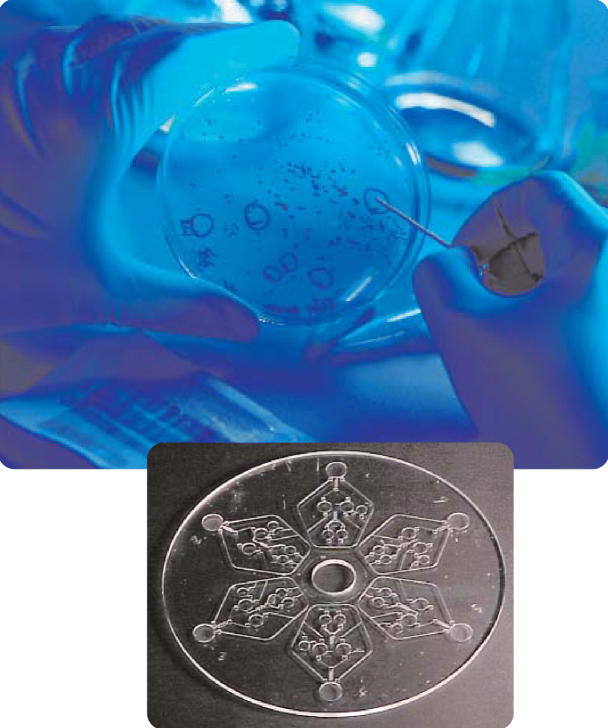# The NIH ENDGAME Consortium

**Published:** 2006-07

The NIH has recently established a highly interactive consortium of 11 research groups on Enhancing Development of Genome-wide Association M**e**thods (ENDGAME) to advance the utility of genome-wide association studies. The consortium (funded by the NHLBI, NIEHS, NCI, NHGRI, and NIGMS) brings together expertise in genetics, epidemiology, biostatistics, and bioinformatics to develop and test innovative, informative, and cost-effective study designs and analytical strategies for performing genome-wide association studies on complex diseases. Available resources such as the International Haplotype Mapping (HapMap) data and single nucleotide polymorphism (SNP) discoveries along with improvements in genome technologies have increased the feasibility of genome-wide association studies for complex diseases. However, it has become increasingly apparent that a major barrier to successfully completing these studies is a lack of both appropriate analytical tools and understanding of which study designs and computational methods are most appropriate for particular study scenarios.

The NIEHS is most interested in the development of analytical tools and approaches that would allow identification of environmental components or covariates of complex diseases in genome-wide association studies. Although most common chronic diseases are the result of complex interactions between genes (G) and environmental (E) factors, most analytical approaches adopted for whole genome scans do not incorporate interactive effects with environmental factors. Studies have indicated that failure to account for G × E interactions in complex disease association analyses can decrease the power to find genetic disease loci and underestimate both the genetic and environmental effects of the disease. The NIEHS is therefore co-funding with the NCI two applications in this consortium, led by Dr. Duncan Thomas of the University of Southern California and Dr. Charles Kooperberg of the Fred Hutchinson Cancer Center, that specifically focus on identifying study designs and analytical methods that will enhance the possibility of identifying gene–gene and gene–environment interactions. All strategies and tools developed through this consortium will be made available to the entire scientific community. The long-term goals of ENDGAME are to accelerate the identification of genetic susceptibility factors in human disease and the ultimate development of novel and individual disease prevention and treatment strategies through the advancement of genome-wide association study methodologies.

## Contact

**Kimberly A. McAllister, Ph.D.** | mcallis2@niehs.nih.gov

## Figures and Tables

**Figure:**